# Comparison of five commercial anti-SARS-CoV-2 total antibodies and IgG immunoassays after vaccination with BNT162b2 mRNA

**DOI:** 10.5937/jomb0-31475

**Published:** 2021-09-03

**Authors:** Elisa Danese, Martina Montagnana, Gian Luca Salvagno, Matteo Gelati, Denise Peserico, Laura Pighi, Simone de Nitto, Brandon M. Henry, Stefano Porru, Giuseppe Lippi

**Affiliations:** 1 University of Verona, Section of Clinical Biochemistry, Verona, Italy; 2 Pederzoli Hospital, Service of Laboratory Medicine, Peschiera del Garda, Italy; 3 Cincinnati Children's Hospital Medical Center, The Heart Institute, Cincinnati, Ohio, United States of America; 4 University of Verona, Section of Occupational Medicine, Verona, Italy

**Keywords:** BNT162b2 mRNA Covid-19 vaccine, immune response, antibodies, immunoassays comparison, BNT162b2 mRNA Covid-19 vakcina, imuni odgovor, antitela, poređenje imunoodređivanja

## Abstract

**Background:**

Since universal vaccinations represents the most effective strategy to mitigate coronavirus disease 2019 (COVID-19), baseline assessment and post-vaccine monitoring of anti-SARS-CoV-2 neutralizing antibodies are essential to vaccination programs. Therefore, this study aimed to compare data of five commercial anti-SARS-CoV2 immunoassays after administration of an mRNA vaccine.

**Methods:**

Venous blood was collected from three healthcare workers, receiving a double (30 g) dose of BNT162b2 mRNA Covid-19 vaccine (Comirnaty, Pfizer), on the day of the first vaccine dose and then at fixed intervals for the following 2 months. Anti-SARS-CoV-2 neutralizing antibody response was assayed with Roche Total Ig anti-RBD (receptor binding domain), DiaSorin TrimericS IgG (spike trimer), Beckman Coulter IgG anti-RBD, SNIBE IgG anti-RBD and Technogenetics IgG anti-N/S1.

**Results:**

A total number of 45 samples were drawn at the end of the 2-month study period. The Spearman's correlations of absolute anti-SARS-CoV-2 antibodies were always excellent (all p<0.001), comprised between 0.967-0.994. Satisfactory results were also observed when absolute antiSARS-CoV-2 antibodies values of the five methods were compared with the mean consensus value, with correlations always higher than 0.979 (all p<0.001). The agreement of anti-SARS-CoV-2 antibodies positivity versus the consensus median positivity ranged between 0.764 and 1.000 (always p<0.001), but become always >0.900 after readjustment of one assay cutoff.

**Conclusions:**

All the immunoassays evaluated in this study appear suitable for monitoring anti-SARS-CoV-2 neutralizing antibodies response in subjects undergoing mRNA COVID-19 vaccination.

## Abstract (sr)

**Uvod:**

Pošto univerzalna vakcinacija pretstavlja najefikasniju strategiju za zaustavljanje koronavirus oboljenja 2019 (COVID-19) veoma je važno praćenje nakon vakcinacije anti-SARS-CoV-2 antitela koja su va`na za programe vakcinacije. Iz tog razloga ovo proučavanje je imalo za cilj da poredi podatke pet komercijalnih anti-SARS-CoV-2 imunodređivanja nakon davanja mRNA vakcine.

**Metode:**

Uzeta je venska krv od tri zdravstvena radnika koja su primila duplu dozu (30 g) BNT-162b2 mRNA Covid-19 vakcine (Comirnaty, Pfizer) odmah nakon davanja prve doze vakcine a zatim u fiksiranim intervalima u roku od sledeća 2 meseca. Anti-SARS-CoV-2 neutrališuća antitela su određivana sa Roche Total Ig anti-RBD (receptor vezujući domen), DiaSorin TrimericS IgG (spike trimer), Beckman Coulter IgG anti-RBD, SNIBE IgG anti-RBD i Technogenetics IgG antiN/S1.

**Rezultati:**

Nakon 2 meseca uzeto je 45 uzoraka. Spearma - nova korelacija apsolutnih anti-SARS-CoV-2 antitela bila je uvek odlična (svi p<0,001) sa obuhvatom između 0,967-0,994. Zadovoljavajući rezultati su dobijeni između vrednosti apsolutnih anti-SARS-CoV-2 antitela dobijenih sa svih pet metoda kad su upoređivane sa konsenzus vrednostima i korelacijom koja je bila veća od 0,979 (svi p<0,001). Slaganje pozitivnosti anti-SARS-CoV-2 antitela prema konsenzus srednjoj pozitivnosti koja se kretala između 0,764 i 1,000 (uvek p<0,001), bila je uvek >0,900 nakon podešavanja cutoff vrednosti za jedno određivanje.

**Zaključak:**

Sva imunodređivanja koja su procenjivana u ovom izučavanju su pogodna za praćenje anti-SARS-CoV-2 netralizirajućih antitela koja nastaju kod osoba koja su bila vakcinisana sa mRNA COVID-19 vakcinom.

## Introduction

Over 1 year after the severe acute respiratory syndrome coronavirus disease 2 (SARS-CoV-2) emerged in Wuhan and then spread around the world causing the worst pandemic outbreak in several decades [1], vaccination appears the most effective strategy to limit the clinical, societal and economic burdens of coronavirus disease 2019 (COVID-19) [2]. With an unprecedented celerity, modern biomedical research has allowed development of a vast array of vaccines, encompassing more traditional products such as inactivated, attenuated, protein subunit and viral vector vaccines, and more recently a new generation of mRNA vaccines [2]. These novel compounds are mostly made of lipid nanoparticles containing prefusion-stabilized protein-encoding mRNA (mostly encoding SARS-CoV-2 spike protein and its receptor binding domain), which are prevalently administered by intramuscular injection [3]. Once in the muscle, myocytes, antigen presenting cells (APCs), dendritic cells and other immune cells in draining lymph nodes uptake these nanoparticles and mRNA is release into the cytoplasm, where it is efficiently translated into mature spike protein [4]. Either expressed at the cell surface in association with major histocompatibility complex (MHC) or released in the surrounding extracellular space after cell injury, the newly synthesized spike protein is presented to B and T cells, triggering the generation of different classes of antibodies and T cells (especially CD4+ and CD8+ cells), which are expected to elicit a solid humoral and cellular immune response against SARS-CoV-2 spike protein [5].

With the clear understanding that universal vaccinations will likely represent the only reliable means to mitigate the deleterious impact of COVID-19 in the forthcoming period [6], baseline assessment and post-vaccine monitoring of anti-SARS-CoV-2 neutralizing antibody (i.e., a class of immunoglobulins (Ig) specifically targeting and thereby inactivating the spike protein and/or its receptor binding domain (RBD) are now regarded as paradigms for prioritizing vaccine administration and monitoring extent and duration of the humoral immune response [7] [8]. To this end, the in vitro diagnostic market is incessantly making available a vast array of anti-SARS-CoV-2 immunoassays, varying in terms of antibody class detected (i.e., total antibodies, thus including IgG, IgM and IgA, rather than IgG only), antigenic target (entire spike protein, subunits 1 and/or 2, RBD) and analytical techniques (ChemiLuminescent Immuno-Assays (CLIA), Enzyme Linked Fluorescent Immuno-Assays (ELFIA), manual Enzyme Linked Immuno-Sorbent Assays (ELISA, etc.)). Whether all these commercial techniques are equally effective for longitudinal monitoring post-vaccination anti-SARS-CoV-2 immune response has important clinical (i.e., risk of infection and developing severe COVID-19 illness) and social (i.e., the potential establishment of so-called »vaccination passports«) consequences, that will need to be constantly addressed and reassessed moving forward [9] [10]. Therefore, this study was aimed at comparing the short-term longitudinal results of five commercial anti-SARS-CoV-2 total antibodies and IgG immunoassays after vaccination with BNT162b2 mRNA Covid-19.

## Materials and Methods

This study encompassed the analysis of postvaccine humoral immune response in three healthcare workers (two females, aged 44 and 39 years, and one male, aged 53 years, respectively), who received 30 µg of the mRNA vaccine Comirnaty (Pfizer Inc, NY, USA), followed by a second 30 µg dose of this same mRNA vaccine 3 weeks later. Venous blood samples were drawn by venipuncture from all three subjects, early in the morning, into evacuated blood tubes containing clot activator and gel (Vacutest, Kima, Padova, Italy). Sampling was scheduled for the morning of the same day when the study subjects received the first mRNA vaccine dose, as well as at different time points afterwards (i.e. 1, 4, 7, 11, 14, 21, 22, 25, 28, 35, 42, 49, 56 and 63 days after the first vaccine dose). Venous blood was separated by centrifugation at 1500×g for 15 min at room temperature within 1 hour from collection, and serum was divided into separate aliquots, stored at -70°C until use. At the end of the study period, the aliquots were thawed, and serum was assayed with five different immunoassays for measurement of total Ig or IgG anti-SARS-CoV-2 antibodies, whose leading technical features are summarized in Table 1. The three study volunteers were also subjected to nucleic acid amplification test (NAAT) of nasopharyngeal swab samples on regular basis (i.e., every 2-3 weeks), for the purpose of ruling out ongoing SARS-CoV-2 infection during the study period. Molecular testing was carried out using the Seegene AllplexTM2019-nCoV Assay (Seegene, Seoul, South Korea), as specified elsewhere [11]. Cumulative results of antibodies testing at the different time points were presented as arbitrary units per mL (AU/mL) or ratio with baseline antibodies value (i.e., (time point level)/(baseline level and/or limit of detection)). After recalibration against the World Health Organization (WHO) International Standard 20/136, the test results of Roche and DiaSorin immunoassays could be converted into WHO binding antibodies units (BAU/mL), as suggested by the manufacturers, whilst arbitrary units were maintained for the other assays which are still only traceable to proprietary standards.

**Table 1:** Technical and analytical features of anti-SARS-CoV-2 antibodies immunoassays used in this study AU, arbitrary units; BAU, binding antibody units; CLIA, ChemiLuminescent ImmunoAssay; Ig, Immunoglobulin; N, nucleocapsid; N/A, RBD, Receptor Binding Domain

Test	Company	Analyzer	Principle	Ig class	Target	Cut-off
Elecsys Anti-SARS-CoV-2 S	Roche	Cobas 8000	CLIA	Total Ig	RBD	≥0.78 WHOBAU/mL
LIAISON SARS-CoV-2 TrimericS IgG	DiaSorin	LIAISON XL	CLIA	IgG	Spike proteintrimer	≥33.8 WHOBAU/mL
ACCESS SARS-CoV-2 IgG II	Beckman Coulter	Access 2	CLIA	IgG	RBD	≥33.8 WHOBAU/mL
MAGLUMI Anti-SARS-CoV-2 S-RBD	SNIBE	Maglumi	CLIA	IgG	RBD	≥1 AU/mL
TGS COVID-19 IgG	Technogenetics	IDS-iSYS	CLIA	IgG	Nucleocapsid/Spike (S1)	≥11.5 AU/mL

Spearman's test was used to assess the correlation of anti-SARS-CoV-2 antibodies values measured after mRNA vaccination with the five different immunoassays, whilst kappa statistics was employed to verify the agreement among anti-SARS-CoV-2 antibody positivity of the five different methods (i.e., positive/negative test results according to the immunoassay specific cutoffs). Correlation and agreement were also calculated versus the mean (consensus) anti-SARS-CoV-2 antibody levels of the five immunoassays obtained at each time point for each of the subjects, and versus the median (consensus) positivity/negativity of the anti-SARS-CoV-2 test results based on immunoassay-specific cut-offs obtained at each time point for each of the subjects, respectively. Positivity was defined as a value exceeding the assay-specific cut-off defined by each manufacturer. Statistical analysis was carried out using Analyse-it (Analyse-it Software Ltd, Leeds, UK). The study protocol was approved by the Ethics Committee of the provinces of Verona and Rovigo (2683CESC; February 16, 2021).

## Results

All NAATs for SARS-CoV-2 RNA detection were consistently negative in the three study subjects, nor did clinical signs or symptoms of COVID-19 develop, such that active SARS-CoV-2 infection was excluded throughout the study period.

**Figure 1:**
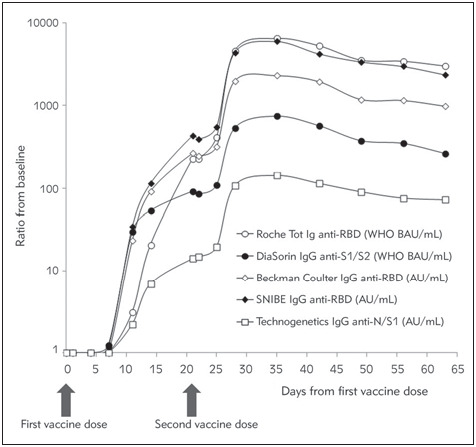
Overall kinetics of anti-SARS-CoV-2 antibodies following BNT162b2 mRNA Covid-19 vaccination (Comirnaty, Pfizer). Values are shown as mean of the three individual values Ig, immunoglobulin; N, nucleocapsid; RBD, receptor binding domain; S, spike protein; Tot, total

A total number of 45 samples (15 for each of the three study subjects) were drawn at the end of the 2-month study period. The cumulative kinetics after mRNA vaccination of the ratio with baseline antibodies values is shown in Figure 1. The levels of the antibodies measured with all the five immunoassays started to raise ~1 week after receiving the first mRNA vaccine dose, displaying a nearly exponential increase up to the 3^rd^ week, when the curve tended to flatten. After the second mRNA vaccine dose, at day 21, the levels of the antibodies measured with all the five immunoassays exhibited a second sharp increase, up to day 30, when the curve tended to flatten again. From day 35 onward, the levels of antibodies measured with all the five different immunoassays displayed a gradual decline, though their values remained considerably higher than the baseline at the end of the study period. Specifically, the fold increase from baseline at day 63 after the first mRNA vaccine dose was still 3.03×10^3^ for Roche Tot Ig anti-RBD, 0.26×10^3^ for DiaSorin TrimericS IgG, 0.98×10^3^ for Beckman-Coulter IgG anti-RBD, 2.37×10^3^ for Snibe IgG anti-RBD and 0.07×10^3^ for Technogenetics IgG anti-N/S1, respectively.

The Spearman’s correlations of absolute (i.e., AU/mL) anti-SARS-CoV-2 antibodies values obtained with the five different methods are shown in Table 2. The correlations were always excellent (all p<0.001), and comprised between 0.967–0.994. Similar satisfactory results were observed when the absolute anti-SARS-CoV-2 antibodies values obtained with the five different methods were compared with the mean consensus value (Table 3), with correlations always comprised between 0.979–0.986 (all p<0.001). The agreement of anti-SARS-CoV-2 antibodies positivity versus the consensus median positivity is shown in Table 4. Kappa statistics was comprised between 0.764 and 1.000. When the Technogenetics IgG anti-N/S1 immunoassay cut-off used for diagnosing SARS-CoV-2 infection (i.e., 11.5 AU/mL) was replaced with the mean value reported in SARS-CoV-2 negative samples (i.e., 1.7 AU/mL), the agreement with the consensus median of antibody positivity of this technique improved significantly, with kappa statistics increasing from 0.764 to 0.947 and nearby equaling that of the other methods.

**Table 2:** Spearman’s inter-correlation of anti-SARS-CoV-2 antibodies levels in three subjects vaccinated with BNT162b2 mRNA Covid-19 (Comirnaty, Pfizer) and followed-up for 2 months 95% CI, 95% confidence interval; Ig, immunoglobulin; N, nucleocapsid; RBD, receptor binding domain; S, spike protein; Tot, total

Antibodies	DiaSorin TrimericS IgG	Beckman Coulter IgG anti-RBD	SNIBE IgG anti-RBD	Technogenetics IgG anti-N/S1
Roche Tot Ig anti-RBD	0.976 (95% CI, 0.956–0.987) p<0.001	0.977 (95 CI, 0.958–0.987) p<0.001	0.987 (95% CI, 0.976–0.993) p<0.001	0.994 (95% CI, 0.988–0.997) p<0.001
DiaSorin TrimericS IgG	–	0.973 (95% CI, 0.952–0.985) p<0.001	0.967 (95% CI, 0.940–0.982) p<0.001	0.979 (95% CI, 0.962–0.988) p<0.001
Beckman Coulter IgG anti-RBD	–	–	0.984 (95% CI, 0.971–0.991)	0.972 (95% CI, 0.950–0.985) p<0.001
SNIBE IgG anti-RBD	–	–	–	0.986 (95% CI, 0.975–0.993) p<0.001

**Table 3:** Spearman’s correlation vs. the consensus mean of anti-SARS-CoV-2 antibodies levels in three subjects vaccinated with BNT162b2 mRNA Covid-19 (Comirnaty, Pfizer) and followed-up for 2 months 95% CI, 95% confidence interval; Ig, immunoglobulin; N, nucleocapsid; RBD, receptor binding domain; S, spike protein; Tot, total

Antibodies	Consensus mean
Roche Tot Ig anti-RBD	0.979 (95% CI, 0.962– 0.989) p<0.001
DiaSorin TrimericS IgG	0.984 (95% CI, 0.971–0.991) p<0.001
Beckman Coulter IgG anti-RBD	0.984 (95% CI, 0.971–0.991) p<0.001
SNIBE IgG anti-RBD	0.986 (95% CI, 0.975–0.992) p<0.001
Technogenetics IgG anti-N/S1	0.982 (95% CI, 0.968–0.990) p<0.001

**Table 4:** Agreement versus the consensus median positivity of anti-SARS-CoV-2 antibodies positivity in three subjects vaccinated with BNT162b2 mRNA Covid-19 (Comirnaty, Pfizer) and followed-up for 2 months *With cut-off >1.7 AU/mL: 0.947 (95% CI, 0.845–1.049; p<0.001) <br>95% CI, 95% confidence interval; Ig, immunoglobulin; RBD, receptor binding domain; S, spike protein; Tot, total

Antibodies	Kappa statistics vs. consensus median
Roche Tot Ig anti-RBD	0.947 (95% CI, 0.845–1.049) p<0.001
DiaSorin TrimericS IgG	1.000 (95% CI, 1.000–1.000) p<0.001
Beckman Coulter IgG anti-RBD	0.900 (95% CI, 0.766–1.035) p<0.001
SNIBE IgG anti-RBD	0.947 (95% CI, 0.845–1.049) p<0.001
Technogenetics IgG anti-N/S1	0.764 (95% CI, 0.574–0.953)* p<0.001

## Discussion

Owing to the ongoing challenges of producing, delivering, and distributing a sufficient amount of vaccines all around the world [12], both the assessment of individual baseline status of anti-SARS-CoV-2 neutralizing antibody positivity and the monitoring of humoral immune response mounted after vaccination shall be considered essential tools in the current tug of war against COVID-19 [7] [8]. Reliable evidence has now been provided that COVID-19 patients with measurable anti-SARS-CoV-2 antibodies at baseline would not need to receive a conventional full-dose of the vaccine, since a single dose may be already effective to elicit a humoral immune response comparable to that of a two-dose administration in anti-SARS-CoV-2 naïve individuals [13]. This finding has now been confirmed in a kaleidoscope of real-world studies [14] [15] [16], and should hence be a guide for future vaccination programs. Over time, the monitoring of neutralizing anti-SARS-CoV-2 antibody response is paramount, as it has been previously reported that the inter-individual response to vaccination may vary widely (i.e., up to 30%) [17], and that humoral anti-SARS-CoV-2 immunity tends to progressively fade over time [18] [19], thus leading the way to the consideration for administration of additional vaccine boost(s) when the titer of neutralizing anti-SARS-CoV-2 antibodies would fall below a protective limit. Although anti-SARS-CoV-2 neutralizing antibodies titration before and after vaccination would be necessary for fully, though unpractically, optimizing vaccination programs [20], clear evidence has been provided that commercial immunoassays used for this purpose reliably mirrors the humoral response, thus providing trustable data that could be used for deciding the most suitable vaccination plan on an individual basis.

Some important aspects have emerged from the results of our current evaluation of five different CLIAs for assessment of anti-SARS-CoV-2 total Ig or IgG neutralizing antibodies. First, virtually identical kinetics of post-vaccination neutralizing antibodies could be seen with all the methods tested (Figure 1). Such a good agreement has been confirmed by the excellent correlations observed by inter-comparing the absolute results (i.e., AU/mL) obtained with the five methods, as well as by comparing individual assay test results with the »consensus« mean antibody level (Table 2 and Table 3). The correlation coefficients were always highly significant and greater than 0.967. A good agreement has also been found when the test results were compared as categorical variable, as positive/negative according to the assay-specific cut-offs. Except for Technogenetics IgG anti-N/S1, the kappa statistics were always higher than 0.9, though the analysis of this last immunoassay deserves a specific mention. As declared by the manufacturer, Technogenetics IgG anti-N/S1 utilizes magnetic nanoparticles coated with both nucleocapsid (N) and subunit 1 of the spike protein (S1). Therefore, its affinity for anti-SARS-Cov-2 neutralizing antibodies elicited by BNT162b2 mRNA Covid-19 vaccine, which contains mRNA encoding only for the SARS-Cov-2 spike protein (not the nucleocapsid), may be perhaps different compared to that of the other immunoassays which, instead, use solid phase-coated recombinant spike protein or RBD. This could hence explain the lower absolute response of anti-SARS-CoV-2 antibodies values and the worse categorical agreement (i.e., positive/negative) with the other methods. Nonetheless, a good correlation found with the other immunoassays by comparing absolute test results, along with the evidence that the categorical agreement could be considerably improved by replacing the cut-off used for diagnosing SARS-CoV-2 infection with the mean level found in SARS-CoV-2 negative samples (i.e., using 1.7 rather than 11.5 AU/mL), which would lead us to conclude that cutoff redefinition for post-vaccine sample monitoring would be a rather simple task. Moreover, unlike the other assays, Technogenetics IgG anti-N/S1 would seem theoretically more effective to monitor vaccination with inactivated and attenuated vaccines, which will also elicit an anti-SARS-CoV-2 nucleocapsid antibody response. Notably, no attempts to compare the absolute values of anti-SARS-CoV-2 were made in this study, since only two of these have currently provided indication to standardize test results according to the new WHO International Standard 20/136. Therefore, we believe that raw values comparison would be highly misleading and virtually useless at this point in time, at least until the results of all the different anti-SARS-CoV-2 assay available in the market will be aligned to International Standard.

In conclusions, the results of this original study, which is the very first to compare five commercial anti-SARS-CoV-2 total antibodies and IgG immunoassays after mRNA COVID-19 vaccination to the best of our knowledge, would lead us to suggest that the majority of clinically validated immunoassays tests could be suitably used for purposes of monitoring the anti-SARS-CoV-2 neutralizing antibodies response in subjects undergoing administration of mRNA vaccines. Nonetheless, given the importance of such data, commercial anti-SARS-CoV-2 immunoassays should undergo clinical evaluation for the purpose of post-vaccine monitoring prior to implementation or use, to ensure accuracy and validity, and define appropriate population kinetics, mean values, and cut-offs.

*Research funding*: None declared.

*Author contributions*: All authors have accepted responsibility for the entire content of this manuscript and approved its submission.

*Informed consent*: Informed consent was obtained from all individuals included in this study.

*Ethical approval*: The study was reviewed and approved by the local Ethics Committee of Verona and Rovigo (2683CESC; February 16, 2021).

## Conflict of interest statement

All the authors declare that they have no conflict of interest in this work.
